# Characterisation of the selective binding of antibiotics vancomycin and teicoplanin by the VanS receptor regulating type A vancomycin resistance in the enterococci

**DOI:** 10.1016/j.bbagen.2017.05.011

**Published:** 2017-08

**Authors:** C.S. Hughes, E. Longo, M.K. Phillips-Jones, R. Hussain

**Affiliations:** a Diamond Light Source, Harwell Research & Innovation Campus, Chilton, Didcot OX11 0DE, United Kingdom; b Membranes, Membrane Proteins & Peptides Research Group, School of Pharmacy & Biomedical Sciences, University of Central Lancashire, Preston, PR1 2HE, United Kingdom

**Keywords:** GPAs, glycopeptide antibiotics, MROs, multi-resistant organisms, VRE, vancomycin resistant enterococci, NAM, N-acetylmuramic acid, NAG, N-acetylglucosamine, SRCD, synchrotron radiation circular dichroism, CD, circular dichroism, MSRCD, magnetic synchrotron radiation circular dichroism, NMRSD, normalised mean residual standard deviation, LC-MS/MS, liquid chromatography–tandem mass spectrometry, UHPLC, ultra-high performance liquid chromatography, HCD, higher-energy collisional dissociation, Vancomycin, Teicoplanin, Antibiotic resistance, Enterococci, Circular dichroism, Fluorescence

## Abstract

A-type resistance towards “last-line” glycopeptide antibiotic vancomycin in the leading hospital acquired infectious agent, the enterococci, is the most common in the UK. Resistance is regulated by the VanR_A_S_A_ two-component system, comprising the histidine sensor kinase VanS_A_ and the partner response regulator VanR_A_. The nature of the activating ligand for VanS_A_ has not been identified, therefore this work sought to identify and characterise ligand(s) for VanS_A_. *In vitro* approaches were used to screen the structural and activity effects of a range of potential ligands with purified VanS_A_ protein. Of the screened ligands (glycopeptide antibiotics vancomycin and teicoplanin, and peptidoglycan components N-acetylmuramic acid, D-Ala-D-Ala and Ala-D-y-Glu-Lys-D-Ala-D-Ala) only glycopeptide antibiotics vancomycin and teicoplanin were found to bind VanS_A_ with different affinities (vancomycin 70 μM; teicoplanin 30 and 170 μM), and were proposed to bind via exposed aromatic residues tryptophan and tyrosine. Furthermore, binding of the antibiotics induced quicker, longer-lived phosphorylation states for VanS_A_, proposing them as activators of type A vancomycin resistance in the enterococci.

## Highlights

• Vancomycin and teicoplanin (not peptidoglycan) are proposed as activating ligands.
• Binding interaction involves tryptophan and tyrosine.
• Binding results in increased phosphorylation activity for VanS_A_.

## Graphical abstract

**Figure:**
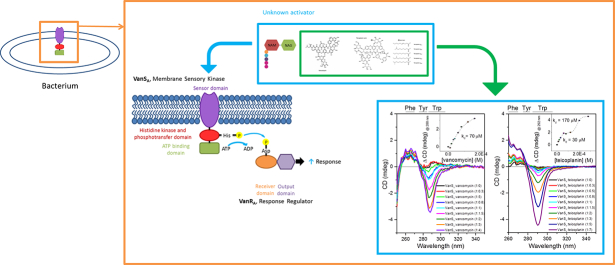

## 1 Introduction

Vancomycin is a tricyclic glycopeptide (molecular weight = 1449) which is used as a first-line treatment for complicated diseases including skin and blood infections, endocarditis, bone and joint infections and meningitis in addition to infections caused by methicillin-resistant *Staphylococcus aureus* [1]. Vancomycin and related glycopeptide antibiotics (GPAs) target transpeptidase and transglycosylase activities by binding to the C-terminal D-Ala-D-Ala dipeptide of the muramyl pentapeptide of peptidoglycan precursor Lipid II which inhibit the final steps of bacterial cell wall biosynthesis. High affinity hydrogen bonds are formed between vancomycin and peptidoglycan precursors [2], [3], [4], sequestering the peptidoglycan precursors and physically inhibiting transpeptidase and transglycosylase cross-linking for the formation of mature peptidoglycan cell walls [4], [5], [6], [7], [8], [9]. Crosslinking of the growing peptidoglycan, formation of glycan chains and incorporation of peptidoglycan precursors is prevented leading to osmotic shock and cell lysis [6], [7], [9].

Using model cell wall precursor peptides such as *N*-acetyl-D-Ala-D-Ala, binding studies in aqueous solution have established that for many glycopeptides, the formation of entropically favourable asymmetric, back-to-back homodimers of the antibiotic [10], [11], [12] is mediated by sugar-sugar recognition [12], [13]. Although vancomycin dimerisation is generally considered to be in a back-to-back configuration, crystal structural information of vancomycin in complex with *N*-acetyl-d-alanine revealed the existence of both back-to-back and face-to-face dimers, though the relative importance of each these configurations in antimicrobial action and affinity remains to be established [14]. Dimerisation is suggested to enhance the activity of the antibiotic, and this is supported by *in vitro* studies showing dimerisation and binding of D-Ala–D-Ala are generally cooperative phenomena [15]. It has been proposed that the dimer binds two adjacent cell wall precursors and that binding to D-Ala–D-Ala at one site on the glycopeptide dimer anchors the dimer to the cell wall and thereby enhances the second binding event through the chelate effect resulting in a stabilised complex [15]. Molecular dynamic simulations and free energy calculations agree, favouring peptidoglycan-glycopeptide interactions rather than direct membrane binding of glycopeptides [16]. Surface plasmon resonance and other studies revealed a k_d_ value for vancomycin binding of 1.32 μM [17], 2.7 μM [18] and 3.39 μM [19], indicative of moderate binding. In the case of the lipophilic glycopeptides such as teicoplanin which do not dimerise but do target the C-terminal D-Ala-D-Ala dipeptide of peptidoglycan [20], [21] with greater potency than vancomycin demonstrated *in vitro* and *in vivo* [22], [23], [24], anchorage into the membrane occurs via the lipid moiety and this anchorage positions the antibiotic at its site of action at the cell surface resulting in an increased effectiveness of the antibiotic [25], [26].

GPAs are considered “last-line” treatments against multi-resistant organisms (MROs). Wide-spread use during the 1970s towards MROs and Gram-positive organisms including enterococci which have innate resistance towards some antibiotics including β-lactams, e.g. penicillin [27], [28], [29], [30] has been attributed a main factor for the development of vancomycin resistant enterococci (VRE) [31], [32]. Resistance to the glycopeptide antibiotics amongst clinical bacteria has emerged and spread across the world at a rapid rate [33].

Of the 6 types of vancomycin resistance [5], [34], type A which was first discovered in *E. faecium* strain BM4147 [34], [35]. The VanA phenotype shows the highest level of inducible resistance towards both vancomycin and teicoplanin and is the most common in the UK [36]. Genes required for type A vancomycin resistance are contained within the *vanA* operon. Expression results in (1) synthesis of low-affinity D-Ala-D-Lac for dipeptide incorporation, and (2) removal of high-affinity D-Ala-D-Ala from early and late peptidoglycan precursors resulting in and overhaul of the incorporated peptidoglycan to D-Ala-D-Lac [5], [27], [37]. The alternative peptidoglycan precursor lacks a key hydrogen bond site normally exhibited by the D-Ala-D-Ala dipeptide, resulting in a 1000-fold reduced affinity for vancomycin [38]. This allows the cell to produce mature cross-linked peptidoglycan and to bypass the cytotoxic activities of vancomycin [5], [39], [40].

Furthermore, concerns for the inter- and intra-species spreading of resistance genes due to the encoding of resistance genes on mobile genetic elements [29] increased after the first *in vitro* transfer of type A vancomycin resistance genes from enterococci to staphylococci [32], later confirmed *in vivo* [41], [42], [43].

Expression of the *vanA* operon is controlled by the two-component signal transduction system VanR_A_S_A_ [44], comprised of the signal sensing membrane sensor histidine kinase VanS_A_ and partner response regulator VanR_A_ which is responsible for activating resistance gene expression [5]. Autophophorylation of the cytosolic kinase portion of VanS_A_ and phosphotransfer to VanR_A_ has been demonstrated *in vitro* [45]. VanR ~ P has been demonstrated to have increased affinity for *van* promoters than VanR_A_ [46]. The presence of VanR_A_ ~ P increases transcription of (1) the P_*vanR*_ promoter regulating VanS/VanR expression therefore regulates its own synthesis, and (2) the P_*vanH*_ promoter for the *vanH*_*A*_*A*_*A*_*X*_*A*_ resistance operon expression [47]. The *vanA* operon is constitutively expressed in the absence of VanS_A_ as a result of the production of VanR_A_ ~ P generated from acetyl phosphate and/or cross-talking histidine kinase phosphodonors [47]. Overall, it is suggested that in the absence of inducer, VanS_A_ phosphatase activity negatively regulates VanR_A_ by dephosphorylating VanR_A_ through its phosphatase activity preventing any significant resistance gene expression. In the presence of inducer, VanS_A_ switches from phosphatase to kinase mode, resulting in higher levels of VanR_A_ ~ P and resistance gene expression [48] [47].

The nature of the activating inducer for VanA-type resistance remains unknown. Vancomycin has been suggested as a ligand for type A systems [49], [50] and has recently been shown to bind in type A equivalent systems of actinomycetes [51]. Other proposed ligands include the antibiotic-peptidoglycan precursor complex [52] and the accumulating peptidoglycan precursors as a result of vancomycin mode of action [53], [54]. Here, we describe the use of *in vitro* approaches to further characterise the ligand-induced activities of VanS_A_.

## 2 Materials and methods

### 2.1 Materials

Vancomycin, teicoplanin, N-Acetyl-D-glucosamine, N-Acetylmuramic acid, D-Ala-D-Ala, Ala-D-γ-Glu-Lys-D-Ala-D-Ala all purchased from Sigma-Aldrich (UK). IPTG, n-dodecyl-β-D-maltoside, carbenicillin purchased from Melford chemicals Ltd.

### 2.2 Gene cloning

The vanS gene of *Enterococcus faecium* BM4147/pFD225 possessing the A-type resistance to vancomycin has been described previously [50], [55], [56], [57].

### 2.3 Medium-scale expression and purification of VanS_A_

6 × 1 L of LB was inoculated with 1 ml of started culture, growing cells in the presence of 100 μg/ml carbenicillin at 37 °C to OC_600_ (0.6) before induction of VanS_A_ expression by addition of 1 mM IPTG. After induction for 3 h at 30 °C cells were harvested at 8000 ×* g* (av) for 10 min. Cells were washed in 10 mM Tris–HCl pH 8.0, 10% glycerol, 0.5 mM EDTA and centrifuged at 8000 ×* g* (av) before resuspending in the same buffer and storage at − 80 °C as described in [55], [58]. Thawed cell pellets from 6 L of culture resuspended in 10 mM Tris–HCl pH 8.0, 10% glycerol, 0.5 mM EDTA were lysed by explosive decompression using a Benchtop cell disruptor (Constant Systems Ltd). Cell lysis was centrifuged at 12,000 ×* g* (av) for 40 min at 4 °C to remove cell debris, and the supernatant removed and ultracentrifuged at 131,000 ×* g* (av) for 2 h at 4 °C. The supernatant was removed and the pellet was washed by resuspending in 10 mM Tris–HCl pH 8.0 and 2 mM β-mercaptoethanol and ultracentrifuging at 131,000 ×* g* (av) for 1 h at 4 °C. The was step was repeated a further two times before a resuspending the mixed membranes in 10 mM Tris–HCl pH 8.0 and storage at − 80 °C.

Purification was conducted as described in [55]. Mixed membrane preparations were adjusted to 4 mg/ml for solubilisation in 10 mM HEPES pH 8.0, 20% glycerol, 1% n-dodecyl β-d-maltoside (DDM), 20 mM imidazole pH 7.9, 2 mM β-mercaptoethanol for 4 h at 4 °C with gentle agitation before ultracentrifugation at 131,000 ×* g* (av) for 40 min at 4 °C. Supernatant was incubated for 8 h with Ni-NTA Agarose (Qiagen) pre-equilibrated with 10 mM HEPES pH 8.0, 20 mM imidazole pH 7.9, 20% glycerol, 0.025% DDM. Recovered resin was washed with Wash buffer (10 mM HEPES pH 8.0, 20 mM imidazole pH 7.9, 20% glycerol, 0.025% DDM) through a 30 ml column before eluting with 5 column volumes of Elute buffer (10 mM HEPES pH 8.0, 20 mM imidazole pH 7.9, 20% glycerol, 0.025% DDM), collecting in 1 ml fractions. Protein-containing fractions were pooled and concentrated to 1 ml (Vivaspin®6 (100 kD MWCO)) before dialysing (Spectra/Por® Float-A-Lyzer® G2 (3.5–5 kDa)) into Exchange buffer (10 mM HEPES pH 8.0, 20% glycerol, 0.025% DDM).

The final protein preparation prepared by concentrating protein-containing elute fractions to 1 ml (Vivaspin®6 (100 kD MWCO)) before dialysing (Spectra/Por® Float-A-Lyzer® G2 (3.5–5 kDa)) into Exchange buffer (10 mM HEPES pH 8.0, 20% glycerol, 0.025% DDM).

### 2.4 Western blotting

Western blot performed as described in [55]. Image acquisition using a ChemiDoc™ XRS + (BioRad), and densitometry analysis preformed using ImageJ [59], [60]. Western blots were performed in Transfer buffer (25 mM Tris, 200 mM glycine, 20% methanol) under standard conditions (90 V, 1 h). The protein-blotted polyvinylidene difluoride (PVDF) membrane was blocked overnight in 10% (w/v) milk in 1 × TBST (10 mM Tris–HCl pH 7.5, 100 mM NaCl, 1% (v/v) Tween 20). The membrane was probed with Anti-His_6_ antibody (INDIA) and developed by incubating with SuperSignal™ West Pico Chemiluminescent Substrate (Thermo Scientific) and exposing to film. Image acquisition using a ChemiDoc™ XRS + (BioRad), and densitometry analysis performed using ImageJ [59], [60].

### 2.5 Protein concentration determination using A_280_

Protein concentration determination was carried out using a P-class 330 nanophotometer (Implen). The Beer-Lambert law was applied using the calculated extinction coefficient (ɛ) 45,770 M^− 1^ cm^− 1^ for VanS_A_-His_6_.

### 2.6 Synchrotron radiation circular dichroism (SRCD) and circular dichroism (CD)

SRCD spectroscopy was carried out in a nitrogen-flushed chamber at beamline B23 at the Diamond Light Source Ltd., Oxfordshire as described in [61], [62]. For CD studies, experiments were conducted using a Chirascan-Plus (Applied Photophysics).

Ligand-containing samples were performed by addition of 5-fold molar equivalent of ligand stocks in 10 mM Tris–HCl pH 8.0 (control incubated with equivalent volume of 10 mM Tris–HCl pH 8.0). All samples were incubated at 20 °C for 30 min prior to data collection. All data was analysed using CDApps [63] where the mean residue weight of VanS_A_ was taken to be 113. Unless otherwise stated, all spectra presented are difference spectra where all relevant background buffers, ligands etc. have been subtracted. Data acquired when the HT of the detector (PMT) was equal to or > 600 V were excluded from the analyses. Far-UV measurements (180–260 nm) were commonly collected using 0.5 mg/ml of VanS_A_ with bandwidth of 1 nm and 1 s integration time. Data presented in molar extinction (Δε). Temperature denaturation measurements were collected over the temperatures (20 °C–95 °C, in 5 °C increments) in the absence and presence of ligands. Samples were incubated at the initial 20 °C for 30 min after ligand or solvent addition prior to data acquisition. At each temperature step, reactions were incubated for 2 min prior to data collection (1 scan). A final scan was acquired post-temperature ramp after returning to 20 °C and incubation for 20 min before data acquisition. Data was analysed using CDApps [63] to obtain difference spectra where all controls (buffers, ligands, etc.) had been subtracted. Change in CD (mdeg) at a specific wavelength was transferred to OriginPro® 9 and plotted against the corresponding temperature for fitting using Gibbs-Helmholtz equation derived from Boltzmann distribution [64], [65] sigmoidal two-state denaturation curve to a Boltzmann distribution and the expression modified to include parameters for fitting of thermal denaturation data for the calculation of the melting temperature (T_m_).

Secondary structure estimations of VanS_A_ were performed from data collected in the far-UV region using the CONTINLL algorithm [66], [67], [68] and SMP 56 (43 soluble, 13 membrane) database.

Near-UV measurements (260–350 nm) were collected using 1 mg/ml of protein using 10 mm pathlength cell, 2 nm bandwidth, 1 nm increments and 1 s integration. Titration experiments were conducted as described for standard near-UV measurements, with the modification of measurements collected after the addition of incremental volumes of ligand stock as described previously [69], [70]. Change in CD (mdeg) a specific wavelength was monitored, the values transferred to OriginPro® and plotted against respective ligand concentration (M) and fitted with a hill1 binding [71] or biphasic dose response [72] function to determine the k_d_ for binding.

### 2.7 Magnetic synchrotron radiation circular dichroism (MSRCD)

The natural CD and SRCD of proteins in the far-UV region is sensitive to their conformation adopted in solution as a function of environment such as solvent polarity, temperature, surfactants, salts, pH and ligand binding interactions. In the near UV region the CD of Tryptophan (Trp) and Tyrosine (Tyr) aromatic amino acid residues are sensitive to their local environment and are natural probes that can be used to determine ligand binding interactions [73]. The MSRCD on the other hand is far less sensitive to the protein backbone conformational behaviour and for Trp and Tyr aromatic side-chain local environments [74], [75]. In the near UV region, the dominating MSRCD of Trp compared to that of Tyr has been found recently to be sensitive to ligand binding [76], [77] suggesting the molecular interaction is affecting the rotation of electron charge during excitation hence the magnitude of the MSRCD signal. MSRCD is therefore a very useful spectroscopic technique to determine the intimate involvement of Trp and Tyr at the ligand binding interface.

MSRCD measurements were made in the near-UV region (260–340 nm) at 20 °C in 1 nm increments using a cell of 1 cm pathlength, 1.2 nm bandwidth and 1 s integration time. Three scans were acquired per sample in the presence of a magnetic field (1.4 T, OLIS) (North-South and South-North orientation). Measurements were analysed using CDApps [63] first by determining the buffer subtracted spectra by subtracting appropriate buffers from each component, e.g. North-South (NS) buffer from NS measurements; and South-North (SN) buffer from (SN) measurements. Resulting SN measurements were subtracted from NS measurements for each condition, e.g. SN VanS_A_ (SN buffer_subtracted) – NS VanS_A_ (NS buffer_subtracted) using OriginPro®. Analysed ligands alone were subtracted from the measured spectra, where appropriate.

### 2.8 Fluorescence spectroscopy

Fluorescence spectroscopy was carried out using a Chirascan-Plus CD Spectrometer (Applied Photophysics) using 1 cm pathlength cell (1 cm × 1 cm, 4 mm × 4 mm window) at 23 °C, 5 nm bandwidth and 600 V, 295 nm excitation wavelength to excite the VanS_A_ Trp residues was applied to the sample and emission monitored between 300 and 500 nm in 1 nm increments. Samples incubated at 20 °C for 30 min prior to data collection.

Data analysis performed using CDApps [63] to obtain values which have been buffer subtracted and dilution-corrected for all components (F values) (protein only, ligand only, protein-ligand). F_o_ values were determined by normalisation to the initial value for each condition, followed by calculation of F/F_o_ using OriginPro® 9. Standard deviation (*n* = 3) shown by error bars.

### 2.9 Activity assays

Assays were conducted as per the instructions provided in the R&D Systems Universal Kinase Activity Kit (Catalogue # EA004, R&D), changing the pH to 8.0; therefore all assays were conducted in 25 mM HEPES pH 8.0, 150 mM NaCl, 10 mM MgCl_2_, and 10 mM CaCl_2_. Compatibility checks for the activity of CD39L2 were made in the new conditions [78]. A phosphate standard curve in the new assay conditions was used to determine the conversion factor (CF) for CD39L2 by calculating the slope of OD_620_ × phosphate input (KH_2_PO_4_). This CF was used in later calculations to determine the amount of PO_4_^2 −^ produced in later experiments. For ligand screens, VanS_A_ (0.2 μg) was incubated in the absence and presence of 5-fold molar equivalent of ligands for 20 min prior to the initiation of autophosphorylation by the addition of ATP; after which autophosphorylation was allowed to proceed for 40 min before reactions were stopped by the addition of 30 μl Malachite Green Reagent A (ammonium molybdate in 3 M sulphuric acid) at *t* = 60. 100 μl distilled H_2_O was added to each well, followed by 30 μl of Malachite Reagent B (malachite green oxalate and polyvinyl alcohol) and the colour allowed to develop for 20 min before determining the optical density at 620 nm.

## 3 Results and discussion

### 3.1 Verification of the successful expression and purification of active VanS_A_

**Fig. 1:**
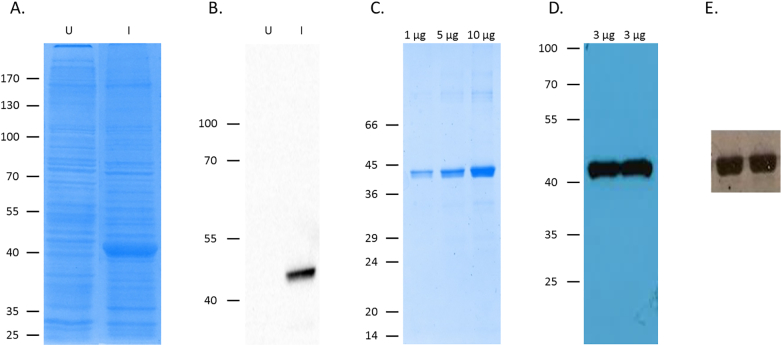
Overexpression, purification and confirmation of active VanS_A_. (A) Coomassie-stained SDS-PAGE gel and (B) Western Blot of mixed *E. coli* membranes from uninduced and induced BL21[DE3] cells harbouring the overexpression plasmid pTTQ. (C) Coomassie-stained SDS-PAGE gel and (D) Western Blot of purified VanS_A_. (E) Autoradiograph of VanS_A_, phosphorylation assay.

**Fig. 2:**
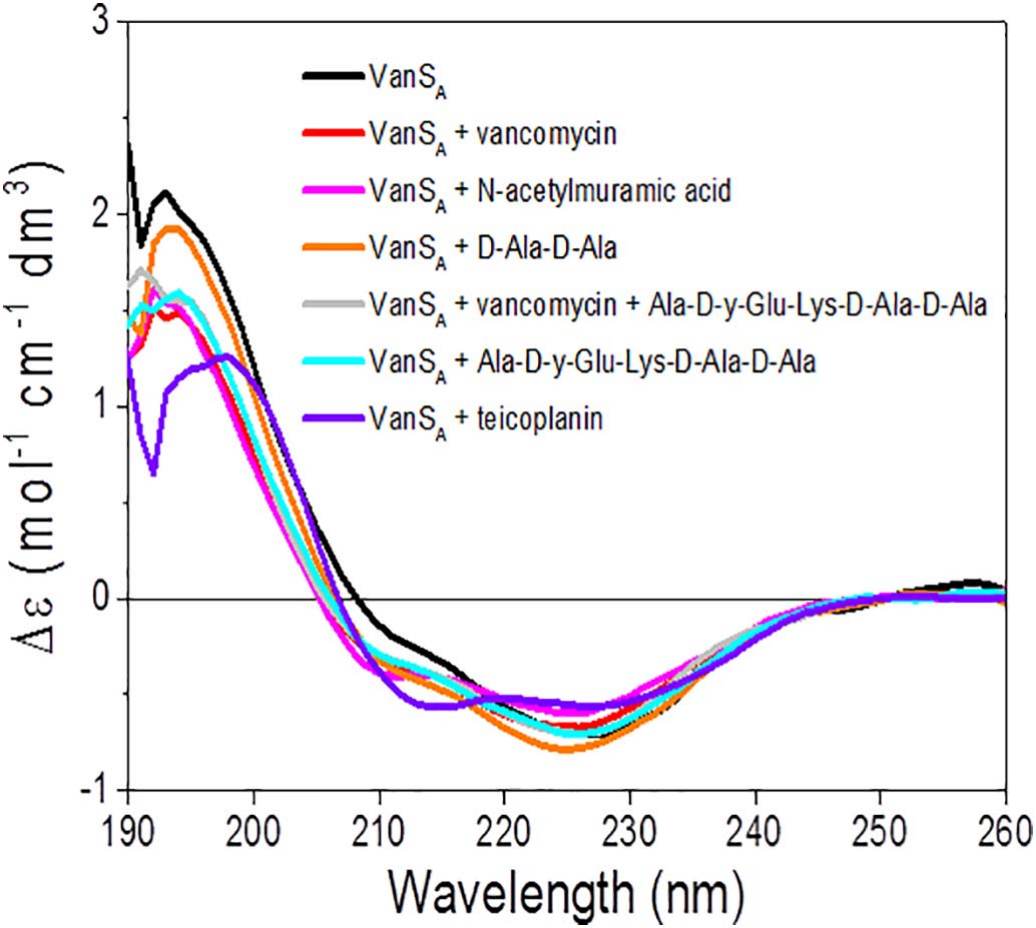
Ligand binding screens of VanS_A_ in the far-UV region (190-260 nm). Difference spectrum of VanS_A_ in the absence and presence of 5-fold molar equivalent of compounds (vancomycin, teicoplanin, N-acetylmuramic acid, D-Ala-D-Ala and Ala-D-y-Glu-Lys-D-Ala-D-Ala).

**Fig. 3:**
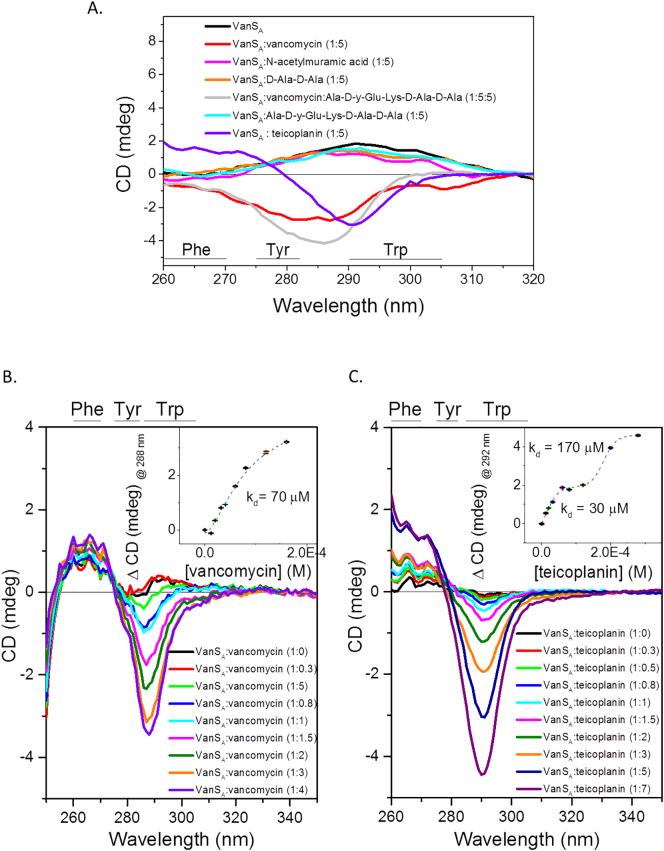
Characterisation of ligand binding in the near-UV region (260–230 nm). Difference spectrum of (A) VanS_A_ in the presence of 5-fold molar equivalent of compounds (vancomycin, teicoplanin, N-acetylmuramic acid, D-Ala-D-Ala and Ala-D-y-Glu-Lys-D-Ala-D-Ala); (B) titration of vancomycin and (C) titration of teicoplanin. Binding constant (k_d_) calculated from change in CD (mdeg) at (B) 286 nm and (C) 292 nm, plotting against respective concentration (M) of GPA and fitting with a (B) Hill1 or (C) BidoseResponse function. Standard deviation (*n* = 4) shown by error bars.

Protein bands (~ 42 kDa) which were revealed using SDS-PAGE as being present only under induction conditions (Fig. 1A and B) were later confirmed by Western blot as VanS_A_ (Fig. 1A and B). VanS_A_ was subsequently purified successfully, its identity confirmed again using SDS-PAGE and Western blot (Fig. 1C, D) in addition to N-terminal sequencing and LC-MS/MS mass spectrometry which confirmed the expressed protein to have the expected amino acid sequence for VanS_A_ (Fig. 1, Fig. 3 [79]). The observed molecular weight for VanS_A_ using SDS-PAGE (~ 42 kDa) was supported by SEC-MALS and LC-MS/MS mass spectrometry with output values of 41,510 ± 930 Da, and 45,764 Da, respectively (Fig. 2, Fig. 3 [79]). Membrane proteins have been reported to migrate anomalously using SDS-PAGE resulting in lower-than-predicted molecular weight outputs [55], [80] and this may account for the lower reported molecular weight by SDS-PAGE and SEC-MALS. Autophosphorylation activity screens confirmed the purification of active proteins (Fig. 1E) which can be used in subsequent ligand binding and activity screens, indicative of no deleterious effect of the presence of detergent on the activity of the protein, similar to results previously reported [55].

Following the production of pure and active protein, biophysical approaches including circular dichroism (CD), magnetic synchrotron radiation circular dichroism (MSRCD) and fluorescence spectroscopy in addition to phosphorylation activity assays were employed to screen a range of candidate compounds (GPAs vancomycin and teicoplanin, and peptidoglycan components) for their effects on VanS_A_ structure and activity.

### 3.2 Assessment of the effects of ligand binding on the conformation and stability of VanS_A_

Ligand binding can induce conformational changes in a protein. Using circular dichroism (CD), changes to the secondary structure (far-UV region, 180–260 nm) and tertiary structure (near-UV region, aromatic region, 260–310 nm) can be monitored [81], [82]. Ligand screens conducted in the far-UV region revealed no significant conformational change for VanS_A_ in the presence of screened compounds in agreement with previous findings [49] with no significant change in the secondary structure estimations (α-helix, 0.04–0.3%; β-strand 0.4–1.6%, turns 0.1–2%, unordered 0.2–0.3%, normalised mean residual standard deviation (NMRSD) is 0.06–0.12).

Differences in the thermal stability of a protein in the absence and presence of ligands can be indicative of binding interactions, therefore thermal denaturation experiments were conducted in the far-UV region as a complementary method for the assessment of binding of the proposed ligands.

A significant increase of the thermal stability for VanS_A_ was observed only in the presence of vancomycin (Table 1; Figs. 5 [79]) and not in the presence of peptidoglycan components suggestive of protein-ligand interactions between VanS_A_ and vancomycin which are favourable to VanS_A_ stability.

**Table 1:** Melting temperature of VanS_A_ in the presence of screened compounds monitored at 222 nm.

Melting temperature (T_m_) of VanS_A_ in the presence of screened ligands (°C)	
VanS_A_	44 ± 0.71
VanS_A_ + vancomycin	79 ± 4.04
VanS_A_ + N-acetylmuramic acid	44 ± 0.63
VanS_A_ + D-Ala-D-Ala	48 ± 4.07
VanS_A_ + vancomycin + Ala-D-γ-Glu-Lys-D-Ala-D-Ala	44 ± 2.23
VanS_A_ + Ala-D-γ-Glu-Lys-D-Ala-D-Ala	51 ± 3.23
VanS_A_ + teicoplanin	47 ± 5.08

### 3.3 Investigation of the role of tryptophan and tyrosine in glycopeptide antibiotic binding by VanS_A_

**Fig. 4:**
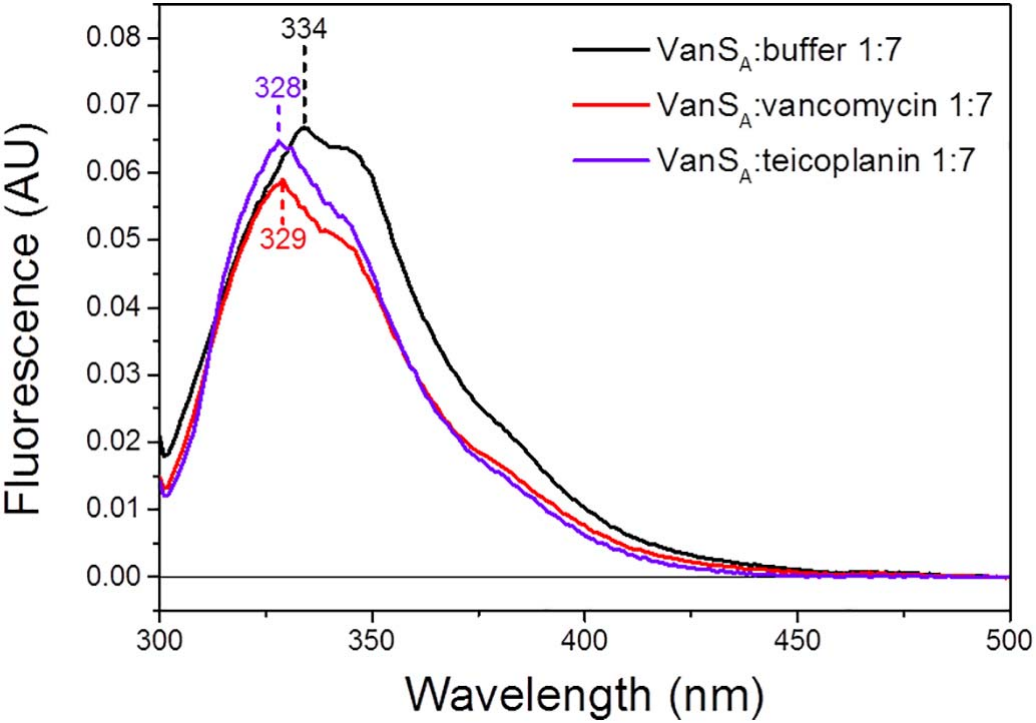
Fluorescence emission spectra of VanS_A_ in the absence and presence of antibiotics equivalent ratio 1:7 (exc. 295 nm).

Next, screens were conducted in the near-UV region to determine the effects of the presence of screened ligands on the local tertiary structure of VanS_A_, specifically screening the aromatic residues of VanS_A_. Qualitative studies revealed changes to the tyrosine/tryptophan region only in the presence of vancomycin and teicoplanin (Fig. 3A) in agreement with previous findings for vancomycin [49] and the first *in vitro* demonstration of binding by teicoplanin. Furthermore, results disregarded peptidoglycan components screened as binding ligands since no significant changes were observed (Fig. 3A). Titration studies demonstrated differences in affinity for each antibiotic. In the case of vancomycin, fitting of the Hill 1 function, a simple sigmoidal function which is used to describe the cooperative occupation of binding sites on a molecule by a ligand as a function of ligand concentration [71], showed binding occurred by a one-step process of moderate affinity (70 μM) (Hill coefficient = 1.65 ± 0.84) (Fig. 3B). Conversely, teicoplanin was found to bind VanS_A_ by a two-step mechanism (Hill coefficient = 0.97 ± 0.77; 0.977 ± 0.76) (Fig. 3C). Fitting was performed using a Bi Dose Response function which is a sigmoidal function monitoring two-step changes induced by exposure to increasing concentration of ligand [72]. The proposed differences in the mechanism of action for each antibiotic [25] could explain these observed differences in mechanism. Alternatively, as teicoplanin is a mixture of compounds [21] which are present in different proportions [20] and display different potencies [20] the observed affinities could result from the binding of different isoforms by VanS_A_, compared to vancomycin which is present as a single compound. UV absorption (A) of a material is an important factor for CD experiments, demonstrated by application of the Beer-Lambert Law. UV absorption is a complementary spectroscopic technique which can be used to investigate ligand binding interactions. Measurements taken at each titration point in the presence of both GPAs supported CD titration results and the calculated k_d_ (Fig. 4 [79]).

**Fig. 5:**
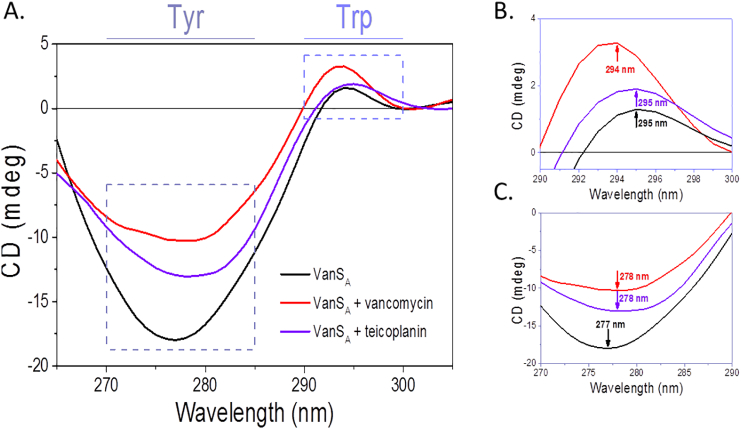
Difference MCD spectra of VanS_A_ in the absence and presence of vancomycin and teicoplanin antibiotics. (A) Displaying 265–330 nm region, (B) tryptophan region (290–300 nm), (C) tyrosine region (270–285 nm). Data smoothed using Savitzky-Golay (10 pts).

To further investigate the roles of tryptophan (Trp) and tyrosine (Tyr) in GPA ligand binding, fluorescence spectroscopy and magnetic synchrotron radiation circular dichroism (MSRCD) techniques were used. These techniques are powerful tools for the investigation of the interactions of the aromatic residues, and can provide information regarding the orientation of the residue of interest (buried or exposed) within the tertiary structure of the protein. In general, the fluorescence emission for proteins is assigned to Trp buried in non-polar environment for λ_max_ < 330 nm and solvent exposed in polar environment for λ_max_ > 330 nm. The shift of λ_max_ as well as the quenching of fluorescence emission in the presence of ligands can be used to monitor binding interactions. Similarly, MSRCD can also be used to probe the interactions of tryptophan and tyrosine aromatic side-chains with ligands. A magnetic field applied parallel to the direction of light propagation enables the distinction of overlapping signals not normally observed using standard CD techniques. MSRCD is most useful when distinguishing signal contributions from tryptophan and tyrosine as the different transitions of the aromatic rings become distinct. In Fig. 4, the λ_max_ of 334 nm for VanS_A_ indicative of the tryptophan being more exposed within the protein tertiary structure was blue shifted to 328 nm and 329 nm upon addition of vancomycin and teicoplanin (GPAs) respectively that was attributed with the Trp less exposed (Fig.4) as a result of antibiotic binding. This was consistent with the MSRCD data that showed analogous shifts of the peak maxima of VanS_A_ (Fig. 5) in the presence of GPAs in both tryptophan and tyrosine spectral regions. The observed red-shifts in the presence of the antibiotics indicated the residues becoming buried as a result of binding suggesting the aromatic residues be important components in the antibiotic ligand recognition.

Previous studies identified a 4-amino-acid motif (DQGW) from the predicted extracellular binding region of VanSsc (equivalent to the type B VanS receptor in soil actinomycetes) to which a modified vancomycin photoaffinity probe bound [51]. Although induction differences have been described for the type A and type B receptors [5], [27], [83], these results nevertheless suggest an important role for tryptophan in vancomycin binding.

### 3.4 Effects of ligand binding on the activity of VanS_A_

**Fig. 6:**
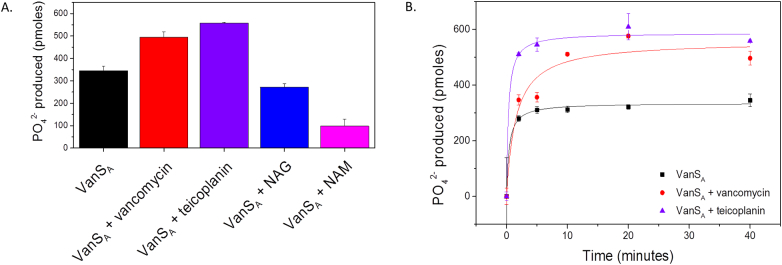
Activity of VanS_A_. (A) In the absence and presence of 5-fold molar equivalent of compounds (vancomycin, teicoplanin, *N*-acetylmuramic acid, *N*-acetylglucosamine). (B) Activity profiles of VanS_A_ in the absence and presence of vancomycin and teicoplanin over 40 min. Standard deviations calculated for each condition (*n* = 3) and plotted as error bars.

Following structural characterisation of ligand binding by VanS_A_, the activity effects of the presence of GPAs vancomycin and teicoplanin, and peptidoglycan components N-acetylmuramic acid (NAM) and N-acetylglucosamine (NAG) were investigated. Results showed significant increases in the activity of VanS_A_ in the presence of GPAs and decreases in activity in the presence of peptidoglycan components (Fig. 6). It is hypothesised that in type A resistance systems, vancomycin binding by VanS_A_ initiates a suppression of phosphatase activity resulting in a net increase in VanR_A_ phosphorylation [27]. Such activities could account for the increased phosphorylation of VanS_A_ observed in this study; however work investigating VanR_A_ phosphorylation in the presence of GPAs would clarify the mechanism.

The observed differences in binding (Fig. 3) and phosphorylation activity (Fig. 6) for VanS_A_ in the presence of teicoplanin may be reflective of the reported potency differences *in vivo* of vancomycin and teicoplanin [84], [85]. By reasoning, the observed tighter binding and increased phosphorylation displayed by VanS_A_ may be a compensatory mechanism which could result in more effective information relay to VanR_A_ and activation of resistance gene expression.

## 4 Conclusion

Here, the characterised binding and activity effects resulting from ligand binding by VanS_A_, the sensor regulating the onset of type A vancomycin resistance in the enterococci, suggest GPAs vancomycin and teicoplanin themselves and not peptidoglycan components are the activating ligands for VanS_A_. Together, this presents a foundation for understanding the activation mechanism for type A resistance which has important clinical implications for ways to prevent the development and spread of resistance, and poses questions regarding the mechanistic differences for the activation of type A and type B resistances.

## Funding

This work was supported by Studentship funding from the University of Central Lancashire and Diamond Light Source.

## Transparency document

Transparency document. Image 1
